# Severe sepsis and sepsis shock secondary to non ventilator associated nosocomial pneumonia. principal features and predictors of outcome

**DOI:** 10.1186/2197-425X-3-S1-A708

**Published:** 2015-10-01

**Authors:** R Zaragoza, N Buceta, S Sancho, C Hurtado, J Camarena, R González, F Puchades, C Martinez, M Cervera

**Affiliations:** Hospital Universitario Dr. Peset, Valencia, Spain

## Introduction

Non ventilator associated nosocomial pneumonia (NVANP) is considered as a rare cause of severe sepsis and septic shock. However its real impact is unknown.

## Objectives

The aims of this study were to describe the principal clinical an epidemiological characteristics of the NVANP severe sepsis and septic shock patients and to describe their clinical and microbiological predictors of outcome.

## Methods

During a year and a half period (January 2013 to July 2014), all NVANP severe sepsis and septic shock consecutively patients in a teaching hospital with sepsis unit were prospectively evaluated, Clinical and microbiological variables were recorded. A univariate analysis was performed to define the factors associated with global mortality (SPSS 20.0). Statistical significance was considered when p value < 0.05.

## Results

Among 950 severe sepsis and septic shock patients activated, 44 of them corresponded to an episode of NAVNP (4.6%). Septic shock was present in 31.8%. The principal place of detection was ER in the 77.3% of the cases. Their mean APACHE II and SOFA score were 19.84 ± 6.96 and 5.27 ± 3.36 respectively. Microbiological documentation was achieved in 38.6%. Among them the main etiologies were: MRSA (22.2%); *Pseudomonas aeruginosa* (22.2%); MSSA (16.6%) and *S. pneumoniae* (16,6%). 15.9% of NAVNP were bacteremic and 84.1% of the episodes received adequate empirical antibiotic treatment (AEAT). Global mortality was 40.9%. In an univariate analysis, presentation as septic shock (64.2 % vs 40.7%, p = 0.03), implementation of SSC early resuscitation bundles (3h) (36.3% vs 42.4%; p = 0.05) and AEAT(32.4% vs 85.7%; p = 0.02) had any impact on prognosis. Neither microbiological documentation (47% vs 37%; p = 0.30) nor bacteremic episodes (57.1 % vs 36.1%, p = 0.27) had any influence on global mortality.

## Conclusions

NAVNP must be considered as a cause of severe sepsis and septic shock and is associated with a poor outcome. Their prognosis is related with the severity of clinical presentation, the implementation of early resuscitation bundles and adequate empirical antibiotic therapy. For these reasons sepsis teams could play a main role in the management of these patiens.

